# Turnover of histones and histone variants in postnatal rat brain: effects of alcohol exposure

**DOI:** 10.1186/s13148-017-0416-5

**Published:** 2017-10-23

**Authors:** Nadia Rachdaoui, Ling Li, Belinda Willard, Takhar Kasumov, Stephen Previs, Dipak Sarkar

**Affiliations:** 1 Department of Animal Sciences, Rutgers Endocrine Research Program, Rutgers, the State University of New Jersey, 67 Poultry Farm Lane, New Brunswick, NJ 08901 USA; 2 Department of Research Core Services, Lerner Research Institute, Cleveland Clinic, Cleveland, OH 44106 USA; 3 Department of Pharmaceutical Sciences, Northeast Ohio Medical University, Rootstown, OH 44272 USA; 4 Cardiometabolic Disease, Merck & Co., Inc, Kenilworth, NJ USA

**Keywords:** Postnatal alcohol exposure, Histone, Turnover, Brain, Post-translational modifications, ^2^H_2_O-labeling, Mass spectrometry

## Abstract

**Background:**

Alcohol consumption during pregnancy is a significant public health problem and can result in a continuum of adverse outcomes to the fetus known as fetal alcohol spectrum disorders (FASD). Subjects with FASD show significant neurological deficits, ranging from microencephaly, neurobehavioral, and mental health problems to poor social adjustment and stress tolerance. Neurons are particularly sensitive to alcohol exposure. The neurotoxic action of alcohol, i.e., through ROS production, induces DNA damage and neuronal cell death by apoptosis. In addition, epigenetics, including DNA methylation, histone posttranslational modifications (PTMs), and non-coding RNA, play an important role in the neuropathology of FASD. However, little is known about the temporal dynamics and kinetics of histones and their PTMs in FASD.

**Results:**

We examined the effects of postnatal alcohol exposure (PAE), an animal model of human third-trimester equivalent, on the kinetics of various histone proteins in two distinct brain regions, the frontal cortex, and the hypothalamus, using in vivo ^2^H_2_O-labeling combined with mass spectrometry-based proteomics. We show that histones have long half-lives that are in the order of days. We also show that H3.3 and H2Az histone variants have faster turnovers than canonical histones and that acetylated histones, in general, have a faster turnover than unmodified and methylated histones. Our work is the first to show that PAE induces a differential reduction in turnover rates of histones in both brain regions studied. These alterations in histone turnover were associated with increased DNA damage and decreased cell proliferation in postnatal rat brain.

**Conclusion:**

Alterations in histone turnover might interfere with histone deposition and chromatin stability, resulting in deregulated cell-specific gene expression and therefore contribute to the development of the neurological disorders associated with FASD. Using in vivo ^2^H_2_O-labeling and mass spectrometry-based proteomics might help in the understanding of histone turnover following alcohol exposure and could be of great importance in enabling researchers to identify novel targets and/or biomarkers for the prevention and management of fetal alcohol spectrum disorders.

**Electronic supplementary material:**

The online version of this article (10.1186/s13148-017-0416-5) contains supplementary material, which is available to authorized users.

## Background

In FASD, both structural and functional defects are produced in the brain [1–3]. Neurons are particularly sensitive to alcohol exposure during critical periods of brain development when neurons form intricate spatial connections and organizations [4]. Mechanisms such as apoptosis, inhibition of proliferation, migration, and differentiation have been implicated in the establishment of alcohol-mediated patterning defects [5, 6]. Animal studies have shown that the neurotoxic action of alcohol during the developmental period reduces the number of hippocampal neurons [7, 8], cortical neurons [9], cerebral granule and Purkinje neurons [10, 11], and hypothalamic neurons [12]. We showed that alcohol increases β-endorphin neuronal death by apoptosis and reduces expression of important neuronal genes such as *Pomc* gene and brain-derived neurotrophic factors (BDNF), which regulate neurogenesis and cell survival in the hypothalamus [12–15]. Accumulation of DNA damage in the absence of repair has been proposed as a key factor in the pathogenicity of alcohol exposure [16]. Alcohol metabolism produces reactive oxygen species (ROS) and acetaldehyde, which have been shown to cause oxidative DNA damage [17, 18] and induce the production of 7,8-dihydro-8-oxo-2′-deoxyguanosine (oxo8dG) DNA lesions [19, 20].

Recent implications of epigenetic mechanisms as mediators of alcohol’s teratogenic effects on the fetus provided insights into the complex characteristics of FASD. The deleterious effects of alcohol during the prenatal and postnatal periods of development correlate with peak periods of epigenetic reprogramming [21, 22]. The existence of such critical developmental periods of vulnerability to the adverse effects of alcohol prompted researchers to study the role of alterations in the epigenome during these early stages of brain development. Garro and colleagues [23] were the first to show that administration of ethanol to pregnant mice on gestation days 9 to 11 resulted in global hypomethylation of fetal DNA, due to a direct inhibitory effect of alcohol on DNMT(s) (DNA methytransferases). We have shown that fetal alcohol exposure (FAE) decreases protein and mRNA levels of histone activating marks (H3K4me3, Set7/9, acetylated H3K9, phosphorylated H3S10) while it increases the repressive marks (H3K9me2, G9a, Setdb1), DNA-methylating enzyme (Dnmt1), and the methyl-CpG binding protein (MeCP2) in the hypothalamus [15, 24, 25]. As DNA damage and repair processes are most likely to play an important role in the alcohol-mediated neurotoxicity in FASD, histone proteins, particularly histone variants have also been shown to play a critical role in these repair processes. For example, deletion of H2Ax or mutation of its Ser139 impairs the recruitment of DNA damage repair proteins, such as BRCA1 and 53BP1 [26, 27]. Most importantly, a HIRA-mediated H3.3 deposition in the DNA damage pathway is also crucial to the maintenance of chromatin integrity and the restoration of the transcriptional activity upon completion of DNA damage repair [28]. Therefore, the interplay between histone variants and chromatin remodeling and accessibility of DNA repair machinery suggests the importance of a sustained availability of histone molecules to ensure that the DNA lesions are faithfully repaired and transcription is resumed for cell function recovery.

Although histone posttranslational modifications (PTM) have been extensively studied in the context of FASD, changes in histone turnover remain unexplored. Herein, we have examined whether alcohol exposure alters histone protein turnover. Previous studies used ^14^C- and ^3^H-radiolabeled amino acids [29], which are complicated in vivo, especially when the proteins of interest have long half-lives. We previously demonstrated that labeled water (e.g., ^2^H) offers advantages in cases where protein synthesis is measured over long periods in free-living animals [30]. It is relatively straightforward to administer labeled water via the drinking bottles; the body will then rapidly generate ^2^H-labeled amino acids [30–32]. We took advantage of the ^2^H-water labeling method to circumvent some obvious problems that are associated with administering a tracer to newborn animals. Quantification of protein labeling is done via standard LC-MS instrumentation generally used in proteomic analyses [31]. Specifically, we investigated the effects of postnatal alcohol exposure (PAE) on the turnover of histone molecules and contrasted two important regions of rat brain, the frontal cortex and the hypothalamus.

## Methods

### Animal procedures

Adult Sprague Dawley rats were purchased from Charles River (Wilmington, MA) and maintained in a controlled environment with a 12-h light/dark cycle at a constant temperature (22 °C). Adult female Sprague-Dawley rats were bred at 2 months of age in our vivarium. At postnatal day 2, rat pups of both sexes were either fed a milk formula containing 11.34% of ethanol yielding a daily ethanol dose of 2.5 g/kg (alcohol-fed, AF) (Bio-Serv, Frenchtown, NJ) or an isocaloric liquid control diet (pair-fed, PF) (Bio-Serv) in which the alcohol calories were replaced by maltose-dextrin. Feedings were conducted at 1000 and 1200 h from postnatal day 2 until postnatal day 6 (PD2-PD6). In a preliminary study, we validated the use of PF as control group by comparing the biological data (BrdU incorporation and γH2A as DNA damage marker) of PF group with ad libitum fed group (AD). The data in Additional file 1: Figure S2 and Additional file 2: Figure S3 shows that the PF and AD data are similar (γH2AX foci/mm, AD-265 ± 46, PF-327 ± 58, *n* = 4–6; *p* > 0.05; BrdU^+^ cells/ mm^2^, AD-1970 ± 173, PF-1775 ± 74, *n* = 4; *p* > 0.05). Hence, we only used PF group as a control group for alcohol treatment. For isotope tracer labeling and proteomic studies, on day 1 of feeding, rat pups (PF and AF) received an intraperitoneal bolus injection of ^2^H_2_O as normal saline and were then fed a milk diet that was enriched with 5% ^2^H_2_O to ensure continuous precursor labeling [30, 33]. Note that the use of the stable isotope (^2^H) labeled water represents an adaptation of studies that previously relied on radioactive (^3^H) labeled water [34]. In some experiments, pups from each experimental group were subcutaneously injected with the free radical spin trap α-phenyl-N-tert-butyl nitrone (PBN) at 100 mg/kg before the morning feeding to investigate the role of ROS production in the effects of PAE on histone turnover. After each feeding, the pups were immediately returned to the litter. In some pups, blood alcohol concentration (BAC) was measured in trunk blood 1 h after the last ethanol feeding. Feeding 11.34% *v*/*v* ethanol increases BAC level to ~ 0.2 g/dl, which is considered to be a moderate BAC. Feeding trials ran for a total of 5 days after which animals were euthanized and samples were quick-frozen. Note that samples were also collected from a subgroup of control animals, which were fed ad lib, and not given any tracer; samples from this group guide the analyses and background corrections. The need for such baseline samples is well known in tracer studies; since stable isotopes are naturally occurring, they generate a background signal that needs to be subtracted from experimental samples [35]. Animal care was performed in accordance with institutional guidelines and complied with National Institutes of Health policy.

### Acid extraction of histone

Brains were collected on PD6 and mediobasal hypothalamus (MBH) and frontal cortex was dissected. The frontal cortex was defined as the tissue extending from the frontal pole caudally to the bregma 3 mm in the pups (from figure 47 to figure 50) [36]. The hypothalamus was sectioned from the posterior portion of the optic chiasm until the end of the mediobasal hypothalamus, from figure 55 to figure 59, according to Ramavhandra and Subrananian [36]. Histones were acid extracted as previously described with some modifications [37, 38]. Briefly, dissected brain tissues were homogenized on ice in Triton Extraction Buffer (TEB: PBS containing 0.5% Triton X 100 (*v*/*v*), protease inhibitors cocktail, and 5 mM sodium butyrate) at 100 mg/ml using a tight-fitting glass Dounce homogenizer by applying 20 strokes and kept on ice for 5 to 10 min. Samples were then centrifuged at 2000 rpm for 10 min at 4 °C. The supernatants were discarded, and the nuclear pellets are washed in half the volume of TEB and centrifuged as above. Histones are then acid extracted in 200 μl of 0.4 N H_2_SO_4_ and incubated overnight at 4 °C [36], then centrifuged at 14.000 g for 15 min at 4 °C and supernatant transferred to a clean 5 ml tube. Two milliliters of cold acetone is then added and incubated at − 20 °C overnight to precipitate histones. Histones are then collected by centrifugation at 14,000 g for 15 min, washed again with acetone then air dried, and resuspended in sterile deionized water. Protein concentration was measured using Bradford assay, and samples were stored at − 20 °C until further analysis.

### Preparation of histone samples for proteomics analysis

Extracted histones were prepared for proteomic analyses as described by [39], with some modifications. Briefly, histones were first derivatized with propionic acid to block unmodified lysines. Briefly, 30 μg of acid-extracted histones were dried down in a SpeedVac, then were dissolved in 15 μl 0.1 M ammonium bicarbonate solution and 10 μl of deionized water. The pH of the samples was adjusted to 8 using concentrated ammonium hydroxide solution and reacted with 20 μl of freshly prepared propionic anhydride/2-propanol solution (1:3 *v*/*v*). The solution was immediately neutralized with 6 μl of concentrated ammonium hydroxide (12 N). If needed, the pH of the solution was adjusted to 8 using additional concentrated ammonium hydroxide. Samples were incubated at 37 °C for 15 min. To remove solvent, the samples were evaporated for 30 min in a SpeedVac. To perform an additional round of propionylation, samples were dissolved in 20 μl of deionized water; pH of the samples were adjusted to 8 using concentrated ammonium hydroxide and propionylated with anhydride/2-propanol solution (1:3 *v*/*v*). Dried samples were then dissolved in 50 μl of 0.1 M ammonium bicarbonate; the pH was adjusted to 8 using concentrated ammonium hydroxide as needed. Histones were digested with 15 μl of 100 ng/mL sequencing grade Tripsin (Promega, Madison, WI) at 37 °C for 6 h. To stop the digestion, 5 μl of acetic acid was added to each sample and then concentrated to < 10 μl using a SpeedVac. After adjusting the pH to 8, propionylation was repeated twice. The volume of each sample was adjusted to 20 μl using 5% trifluoroacetic acid, and 10 μl aliquot was purified and desalted using a C18 Ziptip (Millipore, Darmstadt, Germany). Eluted samples were dried in a SpeedVac and reconstituted in 30 μl of 1% acetic acid for LC-MS analysis.

### LC-MS/MS analysis of proteolytic peptides

Proteomic analysis was carried out by nano-flow LC-MS/MS with a Thermo Acclaim PepMap RSLC C18 nano-column (150 mm × 75 μm, 2 μm, 100 Å) using mobile phase A and B (1% formic acid in water and 0.1% formic acid in acetonitrile, respectively). Tandem mass spectrometry was performed on a Thermo LTQ Oribtrap Elite mass spectrometer (Thermo Scientific, Bremen, Germany) in positive ion mode. Each full scan MS at a resolution of 60,000 (at 400 m/z) was followed by 15 collision induced dissociation (CID) MS/MS (or MS2) scans. Dynamic exclusion was triggered by three consecutive selections within 30 s and last for 90 s.

For protein identification, all of the CID spectra obtained from the mass spectrometer were searched using Sequest which is integrated in Thermo Proteome Discoverer software (Version 1.4) against histone protein extracted from National Center for Biotechnology Information (NCBI) *Rattus norvegicus* reference sequence database. The mass tolerances of the parent and product ions were set at 10 ppm and 1.0 Th, respectively. Trypsin was used as protease with two missed cleavages allowed. Propionylation of K and N-terminus residues were set as fixed modifications. A summary of the histone peptides used to determine histone turnover is presented in Table 1.

**Table 1:** List of histone peptides identified by LC-MS/MS and used to calculate turnover of unmodified and modified histone proteins

Histone	Peptide sequence
H2Aun	_**pr**_ **v**TIAQGGVLPNIQAVLLP**k**_**pr**_ **k**_**pr**_TESHH**k**
H2AK120meK126ac	_**pr**_ **v**TIAQGGVLPNIQAVLLP**k**_**pr**_ **k** ^**me**^TESHH**k** ^**ac**^
H2Bun	_**pr**_ **i**LLPGELA**k**_**pr**_HAVSEGT**k**_**pr**_AVT**k**_pr_YTSA**K**_**pr**_
H2BK9ac	_**pr**_ **i**LLPGELA**k** ^**ac**^HAVSEGT**k**_**pr**_AVT**k**_**pr**_YTSA**K**_**pr**_
H3un	**k**_**pr**_QLAT**k**_**pr**_AAR
H3K1ac	**k** ^**ac**^QLAT**k**_**pr**_AAR
H3K9	**k**_**pr**_STGG**k**_**pr**_APR
H3K9me2	**k** ^**me2**^STGG**k**_**pr**_APR
H3K9me3	**k** ^**me3**^STGG**k**_**pr**_APR
H3K9ac	**k** ^**ac**^STGG**k**APR
H4un	_**pr**_ **g** **k**_**pr**_GG**k**_**pr**_GLG**k**_**pr**_GGA**k**_**pr**_R
H4K5acK9acK13ac	_**pr**_ **gk**_**pr**_GG**k** ^**ac**^GLG**k** ^**ac**^GGA**k** ^**ac**^R
H1	_**pr**_rA**k**_**pr**_AA**k**_**pr**_ **k**_**pr**_STDHP**k**_**pr**_YSDMIVAAIQAE**k**_**pr**_NR
H1.1	_**pr**_sGVSLAAL**k**_**pr**_ **k**_**pr**_SLAAAGYDVE**k**_**pr**_NNSR
H1.4	_**pr**_sGVSLAAL**k**_**pr**_ **k**_**pr**_SLAAAGYDVE**k**_**pr**_NNSR
H1.5	**k**_**pr**_ATGPPVSELIT**k**_**pr**_AVSAS**k**_**pr**_ER
H3.3	**k**_**pr**_SAPSTGGV**k**_**pr**_ **k**_**pr**_PHR
H2Ax	**k**_**pr**_GHYAER
H2Az	_**pr**_aTIAGGGVIPHIH**k**_**pr**_SLIG**k**_**pr**_ **k**_**pr**_GQQ**k**TV

All N-termini and all Lysine residues were propionylated by chemical derivatization. ***me*** methyl, ***ac*** acethyl, *pr* propionyl amide

To quantify 2H incorporation into histones, we manually integrated the areas under each mass isotopomer of tryptic peptides identified in protein database searches. Peptides that cannot be assigned to unique proteins are excluded. The mass isotopic distribution for all selected peptides are quantified as a function of time; the fractional synthesis rate (FSR) is then calculated from the shift in the distribution profile [30, 31].

### GC-MS analysis of plasma water labeling

The ^2^H-labeling of plasma water was determined using GC-MS following the exchange of label with acetone [40]; under alkaline conditions, the ^2^H that is present in water will exchange with hydrogen that is bound to acetone, the enrichment of acetone is then quantified. Briefly, samples are prepared by incubating 5 μL of plasma or known standards in a 2 ml glass screw-top GC vial at room temperature for 4 h with 2 μL 10 N NaOH (Fisher Scientific, Maltham, MA) and 5 μL of acetone (Sigma, St. Louis, MO). The GC-MS is programmed to inject 5 μL of headspace gas from the GC vial in a splitless mode using an isothermal run (Agilent 5973 MS coupled to a 6890 GC oven fitted with an Agilent DB-5MS column), the mass spectrometer is operated in selected ion monitoring (electron impact ionization, m/z 58 and 59, 10 ms dwell time per ion).

### Data analysis and calculation of protein kinetics

**Fig. 1:**
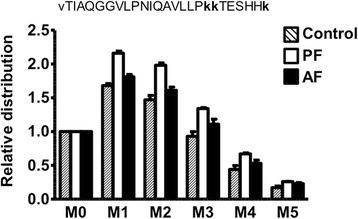
Mass isotopomer distribution profiles of a tryptic peptide from histone H2A. LC-MS analyses were used to determine the isotope profile of the peptide vTIAQGGVLPNIQAVLLP**kk**TESHH**k**; the intensity of the M0 peak (i.e., monoisotopic, m/z 1001.57 with a charge state of 3+) was used to normalize the intensities of the other mass isotopomers. Samples were obtained from control animals (i.e., who did not receive any tracer, shaded bars) and from animals in the respective pair-fed (PF, open bars) and alcohol-fed (AF, solid bars) groups following 5 days of ^2^H_2_O exposure. The mass isotopomer profile in PF animals reflects a more active turnover (i.e., incorporation of ^2^H) as compared to AF animals (which more closely resemble the control animals, representing a reduced turnover with limited incorporation of ^2^H). Data are shown as the mean ± sem, *n* = 3–4 animals per group

As noted above, the first step in the LC/MS analyses is to identify the peptide species. This is done from the accurate mass determination and the fragmentation spectrum. Examples of histone peptide spectra are shown in Additional file 3: Figure S1, and a summary of the histone peptides identified and used for the determination of histone turnover is listed in Table 1. In the case of the peptide vTIAQGGVLPNIQAVLLPkkTESHHk, which is derived from hypothalamic histone H2A, we observed a signal at 1001.57 a.m.u. with a charge state of 3+; this reflects propionyl modifications from the chemical derivatization which are observed at the N-terminal, K119, K120, and K126 residues. The second step in the LC/MS analyses is to determine if the mass isotopomer distribution in a spectrum is shifted from the respective control samples. For example, all proteins have a natural isotopic distribution; they exist as a mixture of slightly different molecular weights. This results from the fact that the various elemental building blocks are not made of homogenous pools, e.g., carbon is present in the body as both ^12^C and ^13^C, hydrogen is present as both ^1^H and ^2^H, etc. A standard approach to better visualize the isotopic distribution of a target analyte involves normalizing the abundance of the heavy isotopes to what is referred to as the “M0” or “monoisotopic” peak, i.e., the analyte which contains only ^12^C, ^1^H, ^14^N, ^16^O, etc. Therefore, in the case of peptide vTIAQGGVLPNIQAVLLPkkTESHHk, we divide each signal by that of the M0 signal (an example of this is shown in Fig. 1).

Isotopic distributions are determined after correction for natural background labeling.

The molar percent enrichment (MPE) of a given peptide is calculated as:

$$ MPE\kern0.3em Mi=\left[ Area\kern0.3em of\kern0.3em Mi/ Sum\kern0.3em of\kern0.3em areas\kern0.3em of\kern0.3em allisotopomers\right]\times 100\%. $$

The total labeling of a peptide is calculated as:

$$ Total\  MPE={MPE}_{M1x1}+{MPE}_{M2x2}+\dots \dots +{MPE}_{Mixi} $$

Since our studies collected samples at a single point in time, we estimated the fractional synthesis rate (FSR) of a given protein using the equation:

$$ FSR\ \left(\%/h\right)= slope of peptide labeling/\left({E}_{water}\times N\right) $$

where “slope of peptide labeling” is the rate of the increase in ^2^H-labeling of the product during ^2^H_2_O administration and *E*_water_ is the steady state enrichment of total body water. *N* is the asymptotic constant number of ^2^H incorporated into an analyzed product, which is calculated based on the number of exchangeable H atoms at C-H sites of a peptide sequence [30, 33].

### Western blot analysis of total histones

Total histone levels were determined by western blot analysis. Whole brain tissue samples were processed for acid extraction of histones and quantification using the Bradford Assay (Bio-Rad Laboratories). Eight to ten micrograms of total histones were run on a 15% SDS PAGE and transferred to PVDF membrane. Membranes were blocked with 5% nonfat dry milk in TBST at room temperature for 2 h then incubated with different primary antibodies for histone proteins at 4 °C overnight. The primary antibodies used were rabbit anti-H2A (cat# 2578, 1:1000, Cell Signaling) and rabbit anti H3 (cat# ab1791,1:1000), rabbit anti-H3.3 (cat# ab62642, 1:1000) and rabbit anti-H2Az (cat# ab4174, 1:1000) from Abcam. We also used rabbit anti-H4 (cat# 07-108, 1/1000, Millipore) and mouse anti-β-actin (cat# CP01-1EA,1:5000, Calbiochem). Membranes were then washed in TBS-T and incubated with the appropriate secondary antibodies conjugated to a horseradish peroxidase followed by chemilumenescence detection using ECL reagents (Thermo Fisher Scientific).

### Immunofluorescence staining for γH2Ax

Twenty millimeters of frozen sections from PF and AF, prefrontal cortex (bregma 4.7 to 3.2 mm), and hypothalamic arcuate nucleus (ARC) (bregma − 1.8 to −4.3) were used for these studies. Sections were fixed with 4% paraformaldehyde and stained overnight at 4 °C with mouse anti-γH2Ax (EMD Millipore, Billerica, MA; 1/250) antibodies. The secondary antibody used was Alexa Fluor 594 donkey anti-mouse (Abcam, Cambridge, MA; 1/500). Sections were mounted with a mounting medium containing 4′,6-diamidino-2-phenylindole (DAPI) to counterstain nuclei (Invitrogen, Waltham, MA). γH2Ax foci were quantified using ITCN (Image-based Tool for Counting Nuclei for ImageJ, NIH, Bethesda).

### Detection of 8-hydroxy 2 deoxyguanosine (8-OHdG)

A competitive ELISA for 8-OHdG was performed using a commercial 8-OHdG ELISA kit (Abcam, Cambridge, MA), following the manufacturer’s instructions. The DNA was purified from frontal cortex and hypothalamus using the DNeasy Blood and Tissue Purification kit (Qiagin, Germantown, MD). DNA quantity and purity were determined using NanoDrop-1000 version 3.7 (Thermo Scientific, Maltham, MA). Enzymatic digestion was performed using nuclease P1 (pH 5.3; Sigma, St. Louis, MO) at 50 °C for 1 h and treated with alkaline phosphatase (pH 8.5; New England BioLabs, Ipswich, MA) at 37 °C for 30 min. Samples were boiled for 10 min and placed on ice for 5 min. DNA hydrolysates were analyzed by ELISA, following the manufacturer’s instructions, and OD was measured at 450 nm using a plate reader (Thermo Scientific, Maltham, MA), and the level of 8-OHdG was determined for each sample from a standard curve.

### BrdU incorporation assay in vivo

In vivo bromodeoxyuridine (BrdU) incorporation was performed to analyze cell proliferation in the brain of rat pups. BrdU (Sigma, St. Louis, MO) (50 mg/kg) was subcutaneously administrated everyday during ethanol feeding from PD4 to PD6 before sacrifice of pups. The brains were then collected and snap frozen in dry ice. Frozen brains from PF and AF from prefrontal cortex (bregma 4.7 to 3.2 mm) and hypothalamic arcuate nucleus (ARC) (bregma − 1.8 to − 4.3) were sectioned at a 20 μm thickness using a Cryostat (Leica CM1900; Leica Microsystems, Buffalo Grove, IL), then fixed with 4% paraformaldehyde then treated with 2 N HCl solution for 30 min at 37 °C, followed by a 10 min incubation with boric acid buffer to neutralize the acid, then washed in PBS. After permeabilization and blocking, sections were incubated with anti-BrdU monoclonal antibody (Sigma, St. Louis, MO; 1:500) overnight at 4 °C, and then with the secondary antibody Alexa Fluor 488 donkey anti-mouse (Abcam, Cambridge, MA; 1:500) for 1 h at room temperature. Sections were mounted with a mounting medium containing 4′,6-diamidino-2-phenylindole (DAPI) to counterstain nuclei (Invitrogen, Waltham, MA). BrdU positive cells were counted using ITCN (Image-based Tool for Counting Nuclei for ImageJ, NIH, Bethesda).

### RNA extraction and real-time polymerase chain reaction

Frontal cortex and hypothalamus were aseptically dissected, and total RNA was extracted from 30 mg tissue samples using RNeasy Mini Kit (Qiagen, Valencia, CA). Total RNA in each sample was quantitated and assessed for quality using the NanoDrop-1000 version 3.7 (Thermo Scientific, Waltman, MA). A ratio of A260/A280 of ~ 2.0 was obtained for all RNA samples. One thousand nanograms per microliter of total RNA from each sample was converted to complementary DNA (cDNA) using high capacity cDNA reverse transcription kit (Applied Biosystems, Carlsbad, CA). RT-PCR was performed at 95 °C for 5 min followed by 40 cycles of 95 °C for 15 s, 60 °C for 30 s, and 72 °C for 40 s in Applied Biosystems 7500 Real-time PCR system (ABI, Carlsbad, CA). The relative quantity of a target mRNA was performed by real-time RT-PCR using SYBR Green (Applied Biosystems, Foster City, CA). Primers used are summarized in Table 2. Measurement of GAPDH and RPL-19 mRNA levels served as internal controls for all experiments. Amplification was performed for 1 cycle of a sequential incubation at 50 °C for 2 min and 95 °C for 5 min, and subsequent 40 cycles of a consecutive incubation at 95 °C for 15 s, 60 °C for 30 s, and 72 °C for 40 s. The individual gene expression value was calculated after normalization to GAPDH and RPL-19.

**Table 2:** List of primers used for Real-Time QPCR analysis

Primers	Sequence
Cycline E1 F	5′- GACAGCTAGCGCGGTGTAG-3’
Cycline E1 R	5′-TTGGAACTCAGACCCGAAGC-3’
Cdk2 F	5′-CTTTGCCGAAATGGTGACCC-3’
Cdk2 R	5′- CCCAGAGTCCGAAAGATCCG-3’
NPAT F	5′- CCTTTTAAGGGCCACCACCTC-3’
NPAT R	5′- AAGGGTAAAGACGCGAGGAC-3’
SLBP F	5′- AGCTCTCCTTCAAACCACCG-3’
SLBP R	5′- ATGGCCTTCAGGCGTTGTAA-3’
H2A F	5′- AAGCTCGTGCAAAAGCGAAG-3’
H2A R	5′- CCTAATGAGGTTGGGGGTGG-3’
H3 F	5′- CGTTGGAGGAGCTTCGTCTT-3’
H3 R	5′- TTGGTTCGGGCCATCTTCTC-3’
H4 F	5′-GCAAGGTCTTGCGGGATAAC-3’
H4 R	5′- GCTCAGTGTAGGTGACTGCG-3’

*F* forward primer, *R* reverse primer, *cdk2* cyclin dependent kinase 2, *NPAT* **n**uclear **p**rotein **a**taxia-**t**elangiectasia; SLBP: **s**tem-**l**oop **b**inding **p**rotein

### Statistical analysis

Statistical analysis of data was performed using Graph Pad Prism software version 5.0 (LA Jolla, CA). Data were analyzed using unpaired student’s *t* test or one way ANOVA with Newman-Keuls post-hoc tests where appropriate. All results are presented as standard error of the mean (SEM). *P* < 0.05 was considered as significant.

## Results

### PAE altered the turnover rates of canonical histones and histone variants

To explore the effects of alcohol exposure on histone turnover in postnatal rat brain, we administered a milk diet containing 11.34% of ethanol (2.5 g/kg per day, alcohol-fed, AF) or an isocaloric liquid control diet (pair-fed, PF) to 2-day-old Sprague Dawley rat pups for 5 days. During the feeding, ^2^H_2_O was added to the milk diet for continuous labeling of newly made proteins. Samples were then collected for determining the protein kinetics via LC/MS.

As expected, there is a change in the relative abundance of the isotopic peaks in animals following 5 days of chronic dosing with the ^2^H_2_O tracer as compared to the spectra that is observed in control animals, which did not receive any ^2^H_2_O; there is a marked difference between the AF and PF groups (Fig. 1). Similar data sets were collected for the various histones that are discussed herein. The fractional synthesis rates (FSRs) are determined from the total change in isotopic labeling of the peptide product as outlined in the “Methods” section. It is important to note that the overall logic which is used here, including the data that are acquired, is virtually identical to that described by Zee et al. [41] with the exception of the shift in isotopic distribution. For example, since Zee et al. [41] administered a more highly substituted precursor (i.e., ^13^C_6_^15^N_2_-lysine) in cultured HeLa cells, they observed a shift in a region of the mass spectra where there is less natural background [40]. Efforts by our group have focused on the use of ^2^H_2_O for reasons noted earlier in regard to the ease of administration in chronic long-term in vivo studies. We also observe a change in the molecular weight (as shown by the shift in isotope distribution in Fig. 1); however, this occurs in a region of the mass spectra where there is more natural background labeling (this is corrected as part of the calculations).

To convert the data contained in Fig. 1 to kinetic rates, e.g., FSR and half-life, one follows a few basic calculations that are outlined in the “Methods” section. The first step involves a calculation of the total enrichment, i.e., subtract the background distribution observed in control animals. The second step is to sum the excess labeling and normalize that against the water labeling; this requires an additional constant factor which is derived from knowledge of the amino acid sequence and the expected equilibration of ^2^H from body water with the respective amino acids [30, 31]. These calculations yield an estimate of the FSR.

**Fig. 2:**
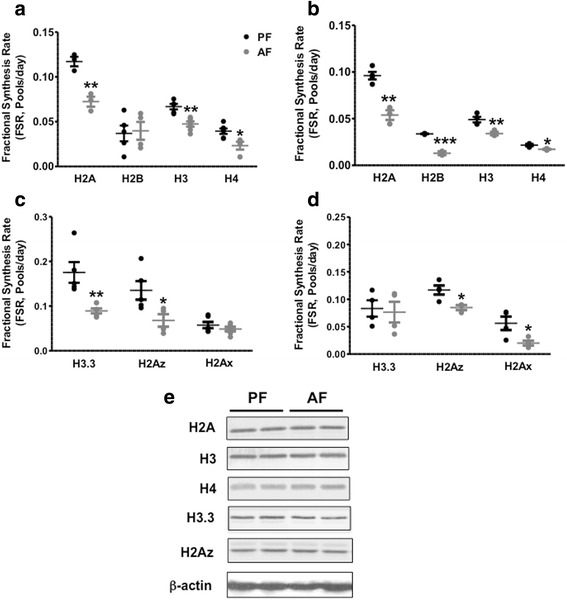
Effects of PAE on the fractional synthesis of replication-dependent canonical histones and histone variants in frontal cortex and hypothalamus of developing rat brain. We measured the incorporation of ^2^H in the canonical histone peptides H2A (**v**TIAQGGVLPNIQAVLLP**kk**TESHH**k**), H2B (**i**LLPGELA**k**HAVSEGT**k**AVT**k**YTSA**K**), H3 (**k**QLAT**k**AAR), and H4 (**gk**GG**k**GLG**k**GGA**k**R) and histone variant peptides H3.3 (**k**SAPSTGGV**kk**PHR), H2Az (aTIAGGGVIPHIH**k**SLIG**kk**GQQ**k**TV), and H2Ax (**k**GHYAER) in frontal cortex (**a**, **c**) and hypothalamus (**b**, **d**) of 6-day-old rat pups following a 5-day alcohol feeding and administration of ^2^H_2_O. Histone Fractional Synthesis Rate (FSR) was calculated as described in the “Methods” section, and data are shown as mean ± SE, *n* = 5–8 per group, *p < 0.05, **p < 0.01, ***p < 0.001, AF vs PF. **e** Western-blot analysis of histone proteins in whole brain of PF and AF animals. The reference protein β-actin is also shown

Our high-resolution accurate mass LC-MS analyses allowed us to identify several histone-derived peptides as well as measure the incorporation of ^2^H. First, we measured ^2^H incorporation in the newly made replication-dependent canonical histones H2A, H2B, H3, and H4. These histones have long half-lives, and their synthesis is tightly linked to DNA replication [42, 43]. Figure 2 clearly shows that H2A followed by H3 has the highest FSR, reflecting faster turnover rates of these histones in both the brain regions studied, the frontal cortex (*F*(3, 10) = 71.37, *p* < 0.0001, Fig. 2a) and the hypothalamus (*F*(3, 10) = 124.8, *p* < 0.0001, Fig. 2b). Figure 2 also clearly shows that PAE significantly decreased the FSR of H2A, H2B, H3, and H4, reflecting a decrease in turnover rates and therefore an increase of the half-lives of these histones in the frontal cortex and the hypothalamus (Fig. 2, Table 3 and Additional file 4: Table S1).

**Table 3:** Summary of the effects of PAE on half-lives of histones in frontal cortex and hypothalamus of rat brain

	Half-life in frontal cortex (d)		Half-life in Hypothalamus (d)	
Histone	PF	AF	PF	AF
H2Aun	5.9 ± 0.3	9.7 ± 0.8**	7.2 ± 0.4	13.1 ± 1.3**
H2AK120meK126ac	6.9 ± 1.1	11.7 ± 3.6	6.8 ± 0.5	10.7 ± 2.3**
H2Bun	27.4 ± 8.9	41.5 ± 15.2	20.5 ± 0.2	65.5 ± 12.1***
H2BK9ac	14.2 ± 4.4	12.8 ± 5.8	9.4 ± 1.5^##^	20.7 ± 2.8** ^#^
H3un	11.0 ± 0.9	14.6 ± 1.1**	14.3 ± 0.9	20.5 ± 1.0**
H3K1ac	7.5 ± 0.4^##^	8.7 ± 0.4^##^	8.9 ± 0.2^#^	17.5 ± 5.9*
H3K9	10.0 ± 0.6	20.2 ± 2.9	19.8 ± 3.0	33.2 ± 1.7
H3K9me2	25.2 ± 6.7	21.4 ± 1.3	18.2 ± 2.8	22.3 ± 3.1
H3K9me3	23.1 ± 5.8	46.1 ± 15.8	38.7 ± 5.7^a^	69.8 ± 4***
H3K9ac	6.4 ± 0.4^#^	12.1 ± 3.3	10.5 ± 2.0^#^	19.9 ± 6.7
H4un	16.2 ± 1.7	29.8 ± 6.3**	28.4 ± 3.4	40.3 ± 1.1*
H4K5acK9acK13ac	8.2 ± 0.7^##^	8.5 ± 0.1^###^	11.5 ± 2.9^#^	14.8 ± 2.6^#^
H1	10.0 ± 1.3	20.4 ± 5.9***	12.3 ± 1.2	19.8 ± 1.2*
H1.1	3.9 ± 0.7	7.9 ± 0.7*	5.7 ± 0.7	10.8 ± 2.2*
H1.4	12.7 ± 2.8	13.9 ± 1.5	11.1 ± 0.6	18.9 ± 0.9***
H1.5	11.3 ± 1.8	20.2 ± 8.2	10.1 ± 0.6	14.5 ± 0.3*
H3.3	4.2 ± 0.6	7.8 ± 0.5**	9.3 ± 2.0	11.3 ± 3.5
H2Ax	12.1 ± 1.6	14.3 ± 1.5	15.1 ± 5.0	39.6 ± 9.1*
H2Az	5.1 ± 0.9	8.6 ± 2.0**	6.0 ± 0.5	8.2 ± 0.5*

Half-lives are calculated from FSR data as described in the “Methods” section. *d* day, *Un* unmodified, *Ac*: acetylated, *me2* di-methylation, *me3* tri-methylation. Data are shown as mean ± SE, *n* = 4 per group, *p < 0.05, **p < 0.01, ***p < 0.001, AF vs PF, #*p* < 0.05, ##*p* < 0.01, ###*p* < 0.001 Nac vs Ac

We also measured the turnover rates of newly labeled replication-independent histone variants (i.e., H3.3, H2Az, and H2Ax). The data shown in Fig. 2c, d and Additional file 4: Table S1 indicates that the histone variant H3.3 has a faster turnover rate in the frontal cortex (Fig. 2c) than in the hypothalamus (Fig. 2d). PAE dramatically decreased H3.3 turnover rate in the frontal cortex with no significant change in the hypothalamus. The turnover rate of H2Az was decreased in both brain regions; however, the turnover rate of H2Ax was not affected in the frontal cortex but significantly decreased in the hypothalamus. These findings indicate that PAE differentially affects the turnover rates of replication-independent histone variants and that differences exist between both brain regions studied.

In addition, we investigated whether these changes in histone turnover are associated with changes in histone abundance; western-blot analysis was used to determine the quantity of total histones in the whole brain of PF and AF animals. Figure 2e clearly shows that PAE had no effect on the abundance of H2A, H3, H4, H3.3 and H2Az histone proteins, suggesting that the total quantity of histones is not affected by PAE and that alcohol’s effect is most likely altering the turnover and therefore the newly made histone pool.

### PAE altered the turnover rates of linker histone H1 and its variants

**Fig. 3:**
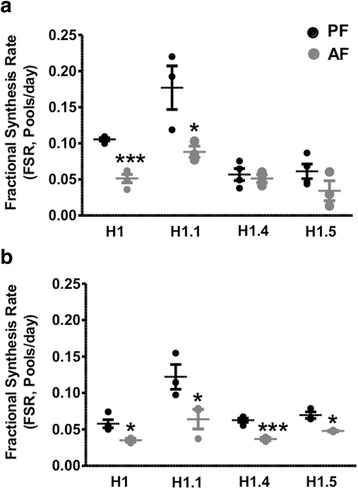
Effects of PAE on the fractional synthesis of linker histone peptides in frontal cortex and hypothalamus of rat brain. We measured the incorporation of ^2^H in the following histone variant peptides H1 (rA**k**AA**kk**STDHP**k**YSDMIVAAIQAE**k**NR), H1.1 (sGVSLAAL**kk**SLAAAGYDVE**k**NNSR), H1.4 (sGVSLAAL**kk**SLAAAGYDVE**k**NNSR), and H1.5 (**k**ATGPPVSELIT**k**AVSAS**k**ER) in frontal cortex (**a**) and hypothalamus (**b**) of 6 days old rat pups following a 5-day alcohol feeding and administration of ^2^H_2_O. Histone Fractional Synthesis Rate (FSR) was calculated and data are shown as mean ± SE, *n* = 4 per group, *p < 0.05, ***p < 0.001, AF vs PF

As their name indicates, linker histones bind to the linker DNA located between two adjacent nucleosomes and provide an additional stabilizing effect on chromatin. We next explored the turnover rates of the linker histone H1 and its variants, H1.1, H1.4, and H1.5 (Fig. 3 and Additional file 4: Table S1). We found that the linker histone H1 and its variant H1.1 had a higher FSR in the frontal cortex than in the hypothalamus. Regardless of the brain region, H1.1 histone had the highest turnover than the other linker histones (*F*(3, 11) = 15.02, *p* < 0.0003 in the frontal cortex, Fig. 3a; *F*(3, 10) = 12.31, *p* < 0.001 in the hypothalamus and 3B). In addition, PAE dramatically decreased the FSR and therefore the turnover rates of H1 and H1.1 in the frontal cortex with no significant changes in H1.4 and H1.5 turnover rates (Fig. 3a). In the hypothalamus, however, the rates of all linker histones were significantly decreased, although to a lesser extent than in the frontal cortex (Fig. 3b).

### PAE altered the turnover rates of modified histones

**Fig. 4:**
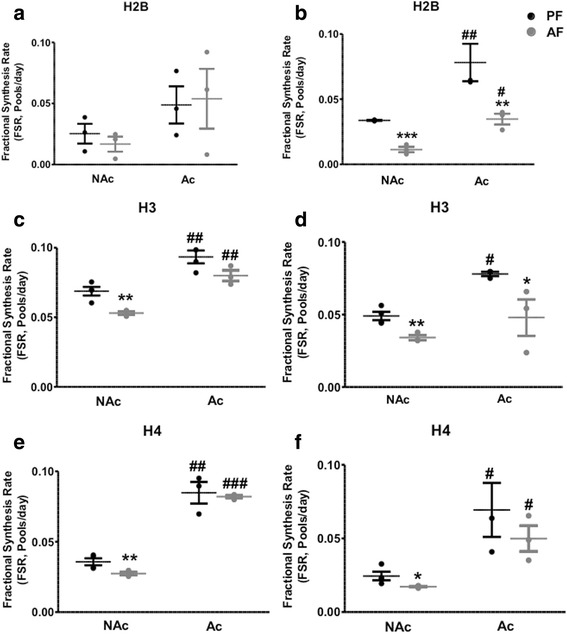
Effects of PAE on the fractional synthesis of non-acetylated (NAc) vs acetylated (Ac) replication-dependent histone peptides in frontal cortex and hypothalamus of rat brain. We measured the incorporation of ^2^H in NAc H2BK9 (**i**LLPGELA**k**HAVSEGT**k**AVT**k**YTSA**k**), H3K1 (**k**QLAT**k**AAR), and H4K5K9K13 (**gk**GG**k**GLG**k**GGA**k**R) and the Ac H2BK9ac (**i**LLPGELA**k**HAVSEGT**k**AVT**k**YTSA**K**), H3K1ac (**k**QLAT**k**AAR), and H4K5acK9acK13ac (**gk**GG**k**GLG**k**GGA**k**R) in the frontal cortex (**a**, **c**, **e**) and the hypothalamus (**b**, **d**, **f**) of 6-day-old rat pups following a 5-day alcohol feeding and administration of ^2^H_2_O. Histone Fractional Synthesis Rate (FSR) was calculated and data are shown as mean ± SE, *n* = 4 per group, *p < 0.05, **p < 0.01, AF vs PF; #*p* < 0.05, ## *p* < 0.01, ### *p* < 0.001 Nac vs Ac

**Fig. 5:**
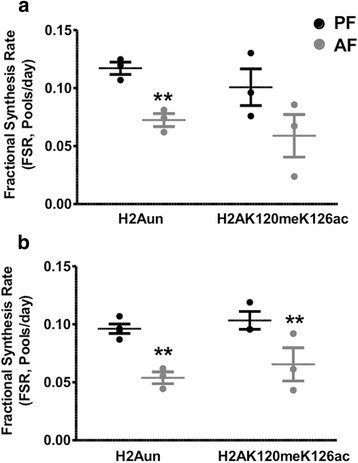
Effects of PAE on the fractional synthesis of doubly modified H2A in frontal cortex and hypothalamus of rat brain. We measured the incorporation of ^2^H in unmodified H2A (H2Aun) and H2AK120meK126ac (**v**TIAQGGVLPNIQAVLLP**kk**TESHH**k**) in the frontal cortex (**a**) and the hypothalamus (**b**) of 6-day-old rat pups following a 5-day alcohol feeding and administration of ^2^H_2_O. Histone Fractional Synthesis Rate (FSR) was calculated and data are shown as mean ± SE, *n* = 4 per group, **p < 0.01, AF vs PF

**Fig. 6:**
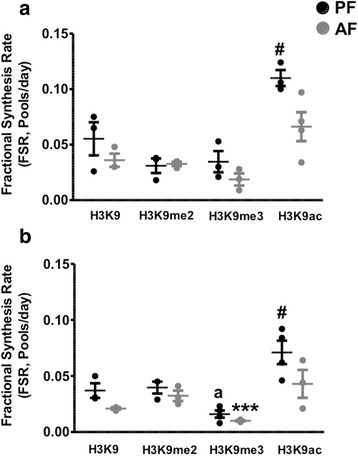
Effects of PAE on the fractional synthesis of H3K9 and its modified peptides in frontal cortex and hypothalamus of rat brain. We measured the incorporation of ^2^H in the histone H3 peptide H3K9 (**k**STGGKAPR**)** and its di-methylated (H3K9me2), tri-methylated (H3K9me3), and acetylated (H3K9ac) isoforms on lysine 9, in frontal cortex (**a**) and hypothalamus (**b**) of 6-day-old rat pups following a 5-day alcohol feeding and administration of ^2^H_2_O. Histone Fractional Synthesis Rate (FSR) was calculated and data are shown as mean ± SE, *n* = 4 per group, ***p < 0.001, AF vs PF, #*p* < 0.05, modified vs unmodified peptides, a *p* < 0.05, H3K9me3 vs H3K9 and H3K9me2

To explore the rates of histone turnover as function of their PTMs, we identified acetylated and methylated peptides of the above aforementioned replication-dependent canonical histones. We determined the relative FSR of the newly labeled modified histone peptides, which then was contrasted to the FSR of the unmodified peptides (Figs. 4, 5, and 6 and Additional file 4: Table S1). Peptide sequences of modified and unmodified H2A, H2B, H3, and H4 analyzed in this study are shown in Table 1.

Figure 4 shows data that were generated from the frontal cortex and the hypothalamus examining the turnover of acetylated peptides identified as H2B, H3, and H4 histones. As reported by others [41, 44], our method confirms the findings that acetylated histones have a significantly faster turnover than the unmodified histones. We found that PAE significantly decreased the turnover rates of unmodified H2B, H3, and H4 in the frontal cortex and the hypothalamus, but had no effect on the acetylated forms in the frontal cortex (Fig. 4a, c, and e) while it decreased the turnover rates of acetylated H2B, H3, and H4 in the hypothalamus (Fig. 4b, d, and f). We were the first to identify a new H2A peptide that was double modified with a methyl group on lysine 120 and an acetyl group on lysine 126 (i.e., H2AK120meK126ac). H2AK120meK126ac has not been reported previously. We calculated the relative FSR of the newly labeled double modified H2A and compared its turnover rate to the unmodified H2A peptide. Interestingly, the turnover rate of H2AK120meK126ac was not significantly different from the unmodified peptide in both brain regions (Fig. 5 and Additional file 4: Table S1). PAE in a similar manner significantly decreased the turnover rates of unmodified as well as doubly modified H2A (Fig. 5 and Additional file 4: Table S1). Furthermore, the analysis of di- and tri-methylated histone H3 revealed that progressively methylated histones have slower turnover rates than the unmodified histones. Figure 6 and Additional file 4: Table S1 show that H3K9me3 had a slower turnover rate than H3K9me2 and H3K9un in the hypothalamus (Fig. 6b). Interestingly, PAE decreased the turnover rates of H3K9un as well as H3K9me3 and had no significant effect on H3K9me2. Histone H3 was shown to be either methylated or acetylated on lysine K9. In this same set of samples, we were able to simultaneously identify an acetylated H3 on lysine 9 (i.e., H3K9ac). Consistent with the findings in Fig. 5, H3K9ac had a faster turnover than the unmodified peptide and the methylated peptides (H3K9me2 and H3K9me3), and in a similar manner was decreased by PAE in both brain regions studied (*F*(3, 8) = 13.00, *p* < 0.0019 in the frontal cortex, Fig. 6a; *F*(3, 10) = 10.99, *p* < 0.0017 in the hypothalamus, Fig 6b).

Since these types of studies generally assume that the kinetics under investigation are described by a single compartment model, and reflect a first-order process, then one can convert the FSR data into protein half-lives using the standard equation half-life = ln 2/FSR [94]. Table 3 presents a comparative summary of the calculated half-lives of the different histone peptides identified in this study.

### PAE induces oxidative DNA damage

**Fig. 7:**
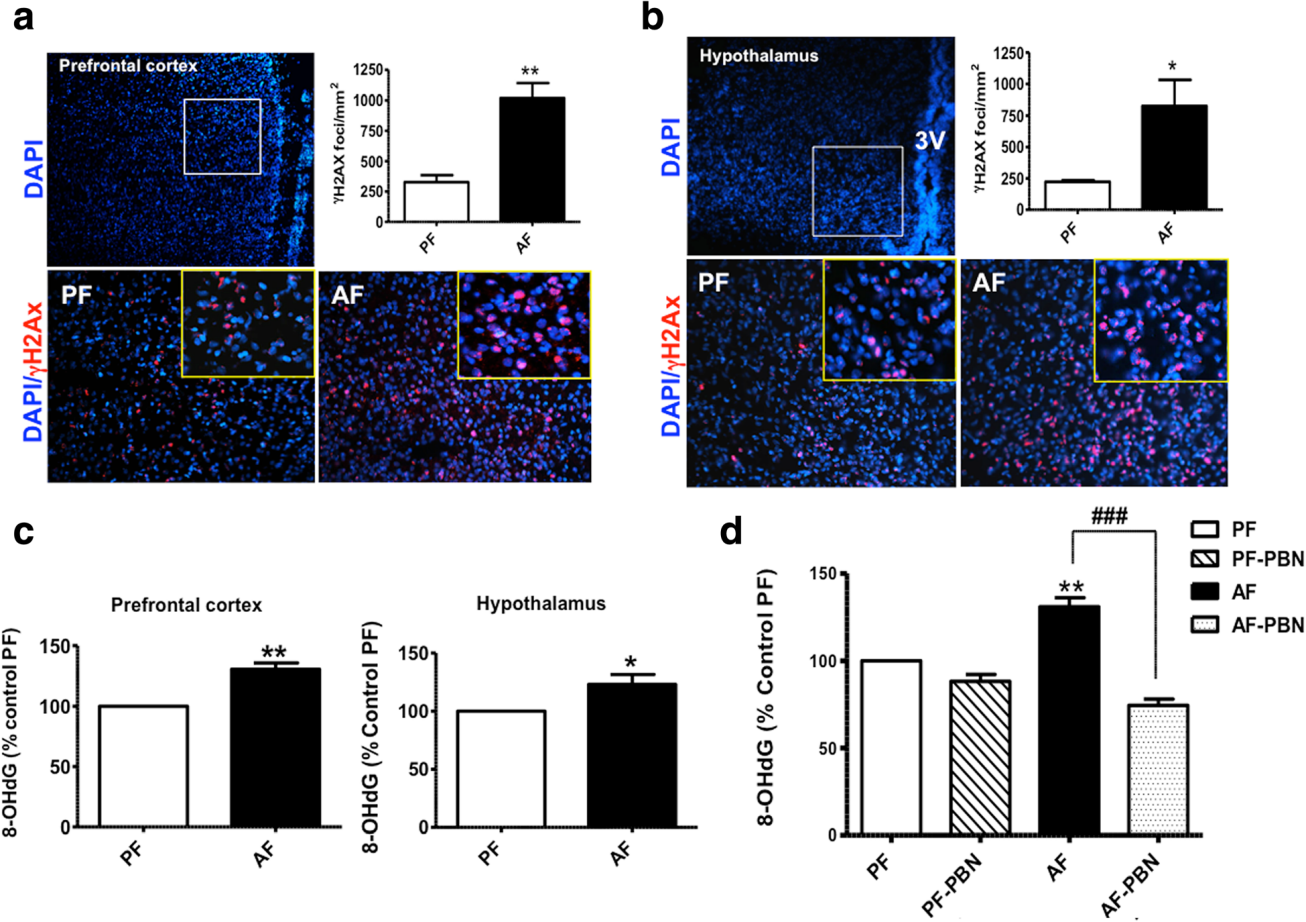
Effects of PAE on oxidative DNA damage in frontal cortex and hypothalamus of rat brain. Frozen sections from frontal cortex (**a**) and hypothalami (**b**) of 6-day-old rat pups were immunostained for γH2Ax (red) and foci were counted using ImageJ (National Institutes of Health, Bethesda, Maryland). Boxed areas in DAPI stained sections indicate the area where representative photographs of prefrontal cortex and hypothalamus were taken. A higher magnification of γH2Ax Foci is shown in the insets. Data are shown as mean ± SE, *n* = 4–6 animals per group done in duplicate. 3V, third ventricle in the hypothalamus. Stains for γH2Ax are shown in red and DAPI in blue. Extracted and hydrolyzed DNA from frontal cortex and hypothalamus (**c**) of 6-days-old PF and AF rat pups was used to confirm the PAE-mediated oxidative DNA damage by measuring 8-hydroxy 2 deoxyguanosine (8-OHdG) using a competitive 8-OHdG ELISA, as described in the “Methods” section. Effects of the free radical spin trap PBN (α-phenyl-N-tert-butyl nitrone) on 8-OHdG formation in AF compared to PF animals was measured in prefrontal cortex (**d**). Data are shown as mean ± SE, *n* = 5–8 done in triplicate

Several studies including ours have shown that alcohol exposure induces ROS production and results in oxidative DNA damage. To investigate whether oxidative DNA damage is involved in the changes of histone turnover observed in response to PAE, we next measured the phosphorylation of histone H2Ax on serine 139, referred to as γH2Ax, an early marker of DNA damage. Figure 7 indicates that PAE significantly increased γH2Ax foci formation in the frontal cortex (Fig. 7a) and the hypothalamus (Fig. 7b) of AF rat pups compared to PF. To confirm these findings, we also measured the formation of 8-hydroxy 2 deoxyguanosine (8-OHdG), a known marker for DNA oxidative damage. Figure 7 shows that PAE significantly increased the formation of 8-OHdG in the frontal cortex and the hypothalamus (Fig. 7c) of AF animals compared to PF. To determine the role of free radicals in the PAE on oxidative DNA damage, we used the spin trap agent PBN. PBN treatment efficiently reduced the formation of 8-OHdG in brain tissue from AF-PBN animals compared to AF animals (Fig. 7d).

### The effect of the free radical spin trap PBN on histone turnover

**Fig. 8:**
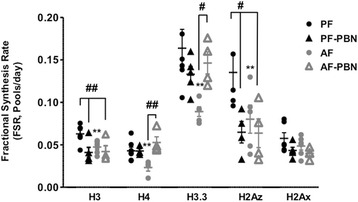
Effects of PBN on fractional synthesis of histones in frontal cortex of PF and AF rat pups. We measured the incorporation of ^2^H in the canonical histone peptides H3 (**k**QLAT**k**AAR), H4 (**gk**GG**k**GLG**k**GGA**k**R) and histone variant peptides H3.3 (**k**SAPSTGGV**kk**PHR), H2Az (aTIAGGGVIPHIH**k**SLIG**kk**GQQ**k**TV), and H2Ax (**k**GHYAER) in frontal cortex of 6-day-old rat pups following a 5-day alcohol feeding and administration of ^2^H_2_O and 100 mg/Kg of the free radical spin trap PBN (α-phenyl-N-tert-butyl nitrone). Histone Fractional Synthesis Rate (FSR) was calculated as described in the “Methods” section, and data are shown as mean ± SE, *n* = 5–8 per group, *p < 0.05, **p < 0.01, AF vs PF, #*p* < 0.05, ##*p* < 0.01, AF-PBN vs PF, AF and PF-PBN

We next investigated the effects of the spin trapping agent PBN on histone turnover. In this study, a subset of rat pups from each experimental group (PF and AF) was subjected to a subcutaneous injection of 100 mg/Kg body weight of PBN once a day. Histones were extracted from prefrontal cortex, digested and analyzed using LC-MS/MS. As shown in Fig. 8, PBN treatment did not inhibit the effects of PAE on H3, H2Az, or H2Ax turnover. However, PBN administration significantly increased H3.3 and H4 turnover rates in AF-PBN animals compared to AF alone. These findings suggest that the free radical spin trap PBN efficiently prevented the effects of PAE on the turnover of H4 and H3.3 histones, but not on H3 and H2Az, indicating a differential effect of this reagent on histones in the brain.

### PAE changed cell proliferation and the expression of cell cycle and histone synthesis genes

**Fig. 9:**
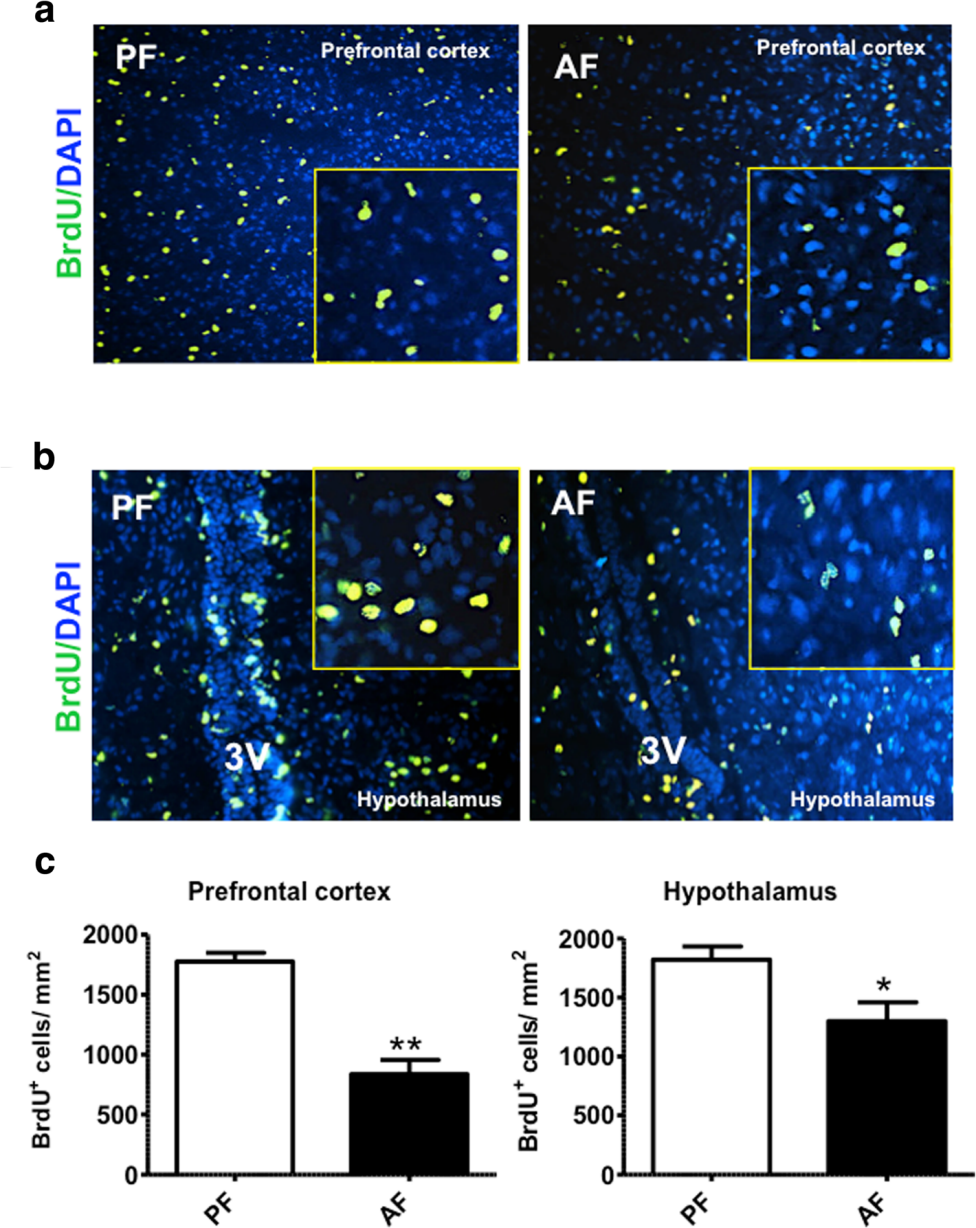
Effects of PAE on BrdU incorporation and cell proliferation in frontal cortex and hypothalamus of rat brain. Representative immunofluorescence images, at 20X, visualized using the fluorescence microscope Nikon Eclipse 2000 (Nikon, Melville, NY) are shown. Higher magnification of BrdU signal is shown in insets. BrdU positive cells were counted in frontal cortex (**a**) and hypothalamus (**b**) of PF and AF animals using ImageJ (National Institutes of Health) and quantifications are shown in (**c**). Data are shown as mean ± SE, *n* = 4–6 animals per group done in duplicates, *p < 0.05, ***p < 0.001, AF vs PF

The PAE-mediated decrease in turnover rates of the replication-dependent canonical histones prompted us to further explore some of the possible underlying molecular mechanisms involved. Since the bulk of canonical histones are synthesized during the S-phase of the cell cycle, we investigated the effects of PAE on cell proliferation using BrdU incorporation and fluorescence microscopy. The number of BrdU-positive cells was counted, and as shown in Fig. 9, PAE significantly decreased the number of proliferating cells in frontal cortex (Fig. 9a, c) and hypothalamus (Fig. 9b, c) of AF animals compared to PF. However, the decrease in cell proliferation was more pronounced in the frontal cortex compared to the hypothalamic region (*p* < 0.0003) (Fig. 9).

**Fig. 10:**
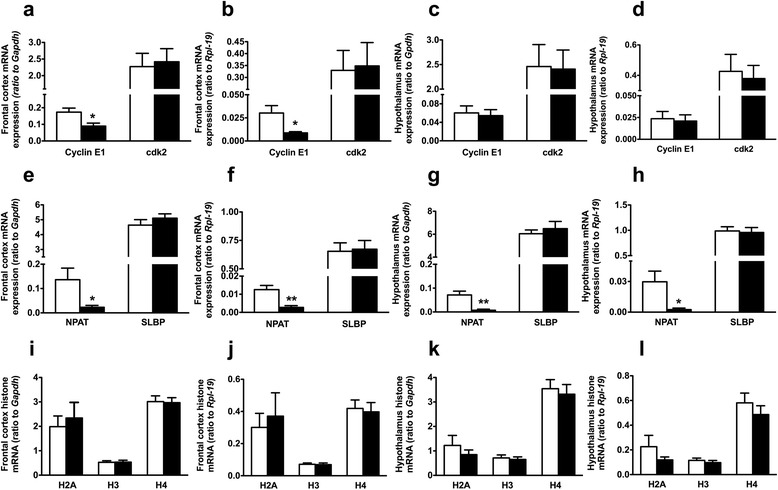
Effects of PAE on gene expression of cell cycle and histone synthesis genes in frontal cortex and hypothalamus of rat brain. Gene expression of cyclin E1 and cdk2, NPAT and SLBP, and histones H2A, H3, and H4 in the frontal cortex (**a**, **b**, **e**, **f**, **i**, and **j**) and hypothalamus (**c**, **d**, **g**, **h**, **k**, and **l**) were measured using RT-qPCR. Data are shown as mean ± SE, *n* = 6 done in duplicate. *p < 0.05, ** *p* < 0.005, AF vs PF

It is well established that the synthesis of canonical histones is tightly coupled to DNA synthesis during the S-phase of the cell cycle. To elucidate some of the molecular mechanisms underlying the decrease in cell proliferation, we next investigated the effects of PAE on the expression of cell cycle genes. PAE significantly decreased the gene expression of cyclin E in frontal cortex (Fig. 10a) and had no significant effect in the hypothalamus (Fig. 10b) of AF rat pups compared to PF. In addition, PAE had no significant effect on cdk2 gene expression (Fig. 10a, b). While PAE dramatically decreased NPAT gene expression in the frontal cortex and the hypothalamus of AF animals, there was no significant change in the expression of SLBP (Fig. 10c, d). Next, we examined the impact of these changes on histone mRNA expression. Figure 10 indicates that PAE had no significant effect on histone mRNA levels in frontal cortex (Fig. 10e) or hypothalamus (Fig. 10f) of AF rat pups compared to PF.

## Discussion

The molecular mechanisms underlying the adverse effects of alcohol exposure on fetal brain remain poorly understood. However, it is becoming clear that epigenetic alterations resulting in changes in gene expression play a key role in the development of FASD. DNA methylation, histone modifications, and non-coding RNA have been suggested as molecular mediators of the adverse effects of FAE [21, 22, 45]. However, one has to recognize that we are still far from having a clear understanding of the complex molecular epigenetic processes involved in FASD. For example, most neuroepigenetic studies have focused on DNA methylation and histone PTMs as the primary mechanisms affecting chromatin structure and gene expression; little is known about the temporal dynamics and turnover of histones. Recently, it has become clear that nucleosomal histone turnover is critical for cell-specific gene transcription, DNA damage repair and recovery, neuronal plasticity, and brain development [46–48]. Herein we have examined whether PAE affects turnover of histone proteins in postnatal rat brain.

Histone turnover has been studied in vivo using ^14^C- and ^3^H-radiolabeled amino acids [29, 49]; in cases where the proteins of interest have long half-lives, this approach is complicated since one may need to continuously administer the labeled precursor. Since ^2^H in body water will rapidly and continuously generate ^2^H-labeled amino acids and since body water has a relatively long half-life, our ^2^H method circumvents a major technical challenge in studies of in vivo kinetics. The analysis of ^2^H-labeled proteins is achieved using standard mass spectrometry-based proteome analyses, which facilitates the identification of specific analytes in complex mixtures [31]. For example, one can examine the kinetics of unmodified as well as modified histones.

The LC-MS approach used herein is similar to that reported by Zee et al. [41], with an obvious difference being the strategy for labeling proteins (note that this difference simply reflects the constraints of working in cell-based vs in vivo models). As shown (Figs. 2, 3, 4, 5, and 6, Table 3 and Additional file 4: Table S1), we were able to determine the kinetics of several histones in vivo in rat developing brain. Analogous to Zee et al. [41], we were also able to quantify the kinetics of some modified histones.

We report that the replication-dependent canonical histones, H2A, H2B, H3, and H4, which were shown to be stable and have long half-lives [42, 43], incorporated ^2^H-labeling during the 5 days exposure of rat pups to ^2^H–water. This indicates that newly synthesized canonical histones are deposited into chromatin. In addition, H2A had the fastest turnover rate compared to H3, H2B, and H4, consistent with previous studies [41, 50]. Several studies have shown that differences in nucleosomal histone turnover are reflective of differences in epigenetic functions and chromatin localizations. For example, histones in promoter regions of active genes have higher turnovers than those in coding regions [51, 52]. We show that PAE decreases turnover rates of canonical histones in frontal cortex and hypothalamus. This indicates that in addition to the effects on DNA and histone epigenetic marks [15, 21, 23–25], alcohol alters histone turnover, which might compromise normal processes of gene expression and cellular functions.

Histone variants, particularly of H2A and H3, are expressed throughout the cell cycle and replace DNA replication-dependent canonical histones. Histone variants have distinct amino acid sequences that can affect the chromatin landscape and nucleosomal dynamics and therefore play critical roles in transcription regulation and other cellular processes such as DNA repair response [53, 54]. Studies have shown that in cortical neurons, the replication-independent histone variants H3.3, H2A.2, and H2Ax replace their canonical counterparts with postnatal age and cellular differentiation [55]. These histone variants are constitutively expressed in a promoter-dependent manner independently of DNA replication. Among the two H3.3 genes identified in the brain (*h3f3a* and *h3f3b*), the expression of *h3f3b* is highly responsive to neuronal stimuli such as glutamatergic receptor activation, GABA receptor inhibition, neurotrophic signaling (e.g., BDNF) and membrane depolarization [46, 56]. Furthermore, deletion of H2Ax or mutation of its Ser139 impairs the recruitment of DNA damage repair proteins, such as BRCA1 and 53BP1 [26, 27]. Most importantly, a HIRA-mediated H3.3 deposition in the DNA damage pathway is also crucial to the maintenance of chromatin integrity and the restoration of the transcriptional activity upon completion of DNA damage repair [28]. In addition, H2A.Z depletion causes an increased sensitivity to ionizing radiation and genomic instability through prevention of BRCA1 binding to sites of DNA damage, indicating that H2A.Z exchange is important for DNA repair by creating an open chromatin structure that facilitates subsequent binding of repair factors [57]. We next sought to analyze turnover of the histone variants H3.3, H2Az, and H2Ax and we show similar findings. However, differential regulations were observed between frontal cortex and hypothalamus. H3.3 had a higher turnover in the frontal cortex, and PAE decreased this turnover rate by ~ 50%, with no effect in the hypothalamus. PAE also significantly decreased the turnover of H2Az. In contrast, the turnover rates of H2Az and H2Ax were significantly decreased in the hypothalamus. This defines a critical aspect of the differential effects of alcohol on distinct brain regions as previously documented [11, 58] and might reflect differential effects of this PAE-mediated reduction in H3.3, H2Az, and H2Ax turnovers on chromatin structure and therefore might influence gene expression.

Linker histones connect nucleosomes and provide additional stability to DNA [59], and more likely contribute to regulation of gene expression. We report that among the replication-dependent histone linkers, H1.1 had the fastest turnover. Studies have suggested that linker histones exhibit preferential genomic distributions and different chromatin affinities [60, 61], which could explain the differences in turnover rates. Histones H1, H1.4, and H1.5 are expressed in all cell types, whereas H1.1 is specifically expressed in testis, spleen, thymus, lymphocytes, and neuronal cells [62], suggesting that linker histones might have different tissue-specific functions. We show that PAE decreases H1 and H1.1 turnovers in the frontal cortex, and H1, H1.1, H1.4, and H1.5 turnovers in the hypothalamus, suggesting differential effects of PAE on linker histones.

In addition, turnover rates of histones vary depending on their PTMs. For example, acetylated histones have faster turnovers than unmodified histones [41, 44], which correlate with localization in the genome. Acetylated histones are predominantly assembled into promoters and enhancers of active genes, whereas repressive methylated histones are present on inactive chromatin regions. We report that acetylated H2B, H3, and H4 have faster turnovers than unmodified histones. H3K9ac had a significantly higher turnover than the unmodified (H3K9) and the methylated peptides (H3K9me2 and H3K9me3). H3K9ac was shown to be associated with promoters of active genes [63]. Most interestingly, we are the first to report a new doubly modified H2A, H2AK120meK126ac, which had a similar turnover rate as the unmodified H2A. Of note, H2AK120meK126ac has not been reported previously and its function is unknown. Studies have suggested that bivalent modifications might exhibit antagonistic actions towards each other, such as in the case of developmental genes, where bivalent modification might have an important role in silencing these genes and keeping them poised for activation [41, 64]. All these findings show that PAE decreases histone turnover regardless of their PTMs. We do not present data on the turnover and recycling of modifications; rather, our findings provide novel information on alcohol’s effects on histone turnover. This implies an effect of alcohol on newly made histone incorporation and assembly into chromatin.

The interplay between histone variants and chromatin remodeling and accessibility of DNA repair machinery suggests the importance of a sustained availability of histone molecules to ensure that the DNA lesions are faithfully repaired and transcription is resumed for cell function recovery. Herein, we demonstrate that PAE, as previously reported [20], increased γH2Ax and 8-OHdG formation indicative of DNA oxidative damage. Interestingly, although the administration of the free radical spin trap PBN efficiently prevented the formation of 8-OHdG in alcohol-fed animals, it did not prevent the effects of PAE on the turnover of all histones studied. Most interestingly, PBN administration almost fully prevented the decrease observed in H3.3 turnover by PAE. Considering the importance of this histone variant in brain development and neuronal plasticity [46, 47], as well as in DNA repair and recovery [28], we believe these findings are intriguing. Further studies are necessary to evaluate the impact of these findings.

We also show that these effects of PAE on histone turnover were associated with decreased cell proliferation. Proper DNA replication during the S-phase of the cell cycle requires that core histones H2A, H2B, H3, and H4 be simultaneously synthesized [65, 66]. Disturbances in DNA or histone synthesis can result in defects in chromosome segregation [67], be deleterious for cell growth [68] and increase DNA damage sensitivity and cytotoxicity [20, 69]. Furthermore, alcohol exposure is known to induce growth arrest through altered expression of cell cycle proteins, inhibition of proliferation, and increased DNA fragmentation [70, 71]. We show that PAE decreased cyclin E1 and NPAT mRNA expression. No significant changes in cdk2, SLBP, or the canonical histone mRNA levels were observed. Replication-dependent histone mRNAs are non-polyadenylated mRNAs with a conserved 3′ stem-loop end structure that is recognized by SLBP, a histone mRNA stabilizing factor, and cleaved by U7 snRNP complex, responsible for the inhibition of the translation of replication-dependent histone mRNAs at the end of the S-phase [72]. Levels of polyadenylated histone mRNAs are low during proliferation [73] and increase during terminal differentiation [74]. Interference with chromatin structure results in the production of polyadenylated histone mRNAs [75]; whether these polyadenylated histone mRNAs are translated into histones is unclear. We suggest that although PAE had no effect on levels of histone mRNA, one cannot exclude the possibility that PAE might interfere with 3′-end processing and/or affect post-translational and chromatin deposition processes of histone proteins.

## Conclusions

These findings demonstrate our ability to measure histone turnover in rat brain using ^2^H_2_O-labeling and mass-spectrometry. We report that PAE induces a global but differential decrease in histone turnover rates in frontal cortex and hypothalamus. Alterations in histone turnover might interfere with histone deposition, chromatin stability, and cell-specific gene expression. We provide novel insights into the effects of PAE on histone turnover in the brain, which might play an important role in the development of the neurological disorders associated with FASD. Further studies exploring cell-specific and function-specific effects of alcohol exposure on histone turnover are necessary for understanding the molecular basis of epigenetic alterations underlying the development of FASD.

## Additional files

Additional file 1: Figure S2. γH2Ax immunostaining in the frontal cortex of AD and PF control animals. Representative immunofluorescence images, at 20X, visualized using the fluorescence microscope Nikon Eclipse 2000 (Nikon, Melville, NY) are shown. γH2Ax foci (red) were counted in the frontal cortex of AD and PF animals using ImageJ (National Institutes of Health). Data are shown as mean ± SE, *n* = 4–6 animals per group done in duplicate. (TIFF 2702 kb)

Additional file 2: Figure S3. BrdU incorporation and cell proliferation in the frontal cortex of AD and PF control animals. Representative immunofluorescence images, at 20X, visualized using the fluorescence microscope Nikon Eclipse 2000 (Nikon, Melville, NY) are shown. BrdU positive cells (green) were counted in the frontal cortex of AD and PF animals using ImageJ (National Institutes of Health). Data are shown as mean ± SE, *n* = 4–6 animals per group done in duplicates. (TIFF 2702 kb)

Additional file 3: Figure S1. MS/MS spectra for rat histone H2A peptide (vTIAQGGVLPNIQAVLLP**kk**TESHH**k**) (**A)** and histone H3 peptide (**k**QLATKAAR) (**B**). Several sequence specific N-terminal (b) and C-terminal (y) ions are identified in these spectra, and these ions confirm the peptides and protein identification. Please note all N-termini and all Lysine residues were propionylated by chemical derivatization. (TIFF 2702 kb)

Additional file 4: Table S1. Summary of the effects of PAE on FSR of histones in frontal cortex and hypothalamus of rat brain. FSR is calculated as described in the “Methods” section. Un: unmodified; Ac: acetylated; me2: di-methylation; me3: tri-methylation. Data are shown as mean ± SE, *n* = 5–8 per group, *p < 0.05, **p < 0.01, ***p < 0.001, AF vs PF, #*p* < 0.05, ##*p* < 0.01, ###*p* < 0.001 Nac vs Ac. (DOCX 16 kb)

## Abbreviations

8-OHdG: 7,8-Dihydro-8-oxo-2′-deoxyguanosine
BAC: Blood alcohol concentration
BDNF: Brain-derived neurotrophic factors
FASD: Fetal alcohol spectrum disorders
FSR: Fractional synthesis rate
LC-MS: Liquid chromatography mass-spectrometry
NPAT: Nuclear protein ataxia-telangiectasia
PAE: Postnatal alcohol exposure
PTM: Posttranslational modification
ROS: Reactive-oxygen species
SLBP: Stem-loop binding protein
