# Supplementary figures and images for: Turnover of histones and histone variants in postnatal rat brain: effects of alcohol exposure
Source: Clin Epigenetics. 2017 Oct 23;9:117. doi: 10.1186/s13148-017-0416-5 (PMC5654083; doi:10.1186/s13148-017-0416-5)

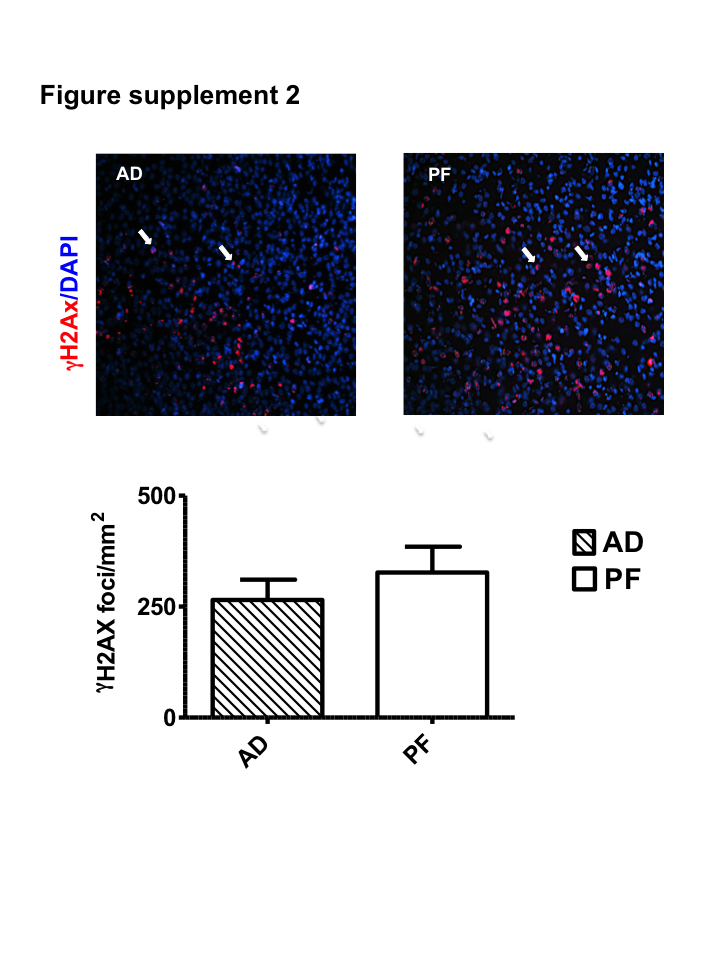

Supplement: Supplementary file 1 — γH2Ax immunostaining in the frontal cortex of AD and PF control animals. Representative immunofluorescence images, at 20X, visualized using the fluorescence microscope Nikon Eclipse 2000 (Nikon, Melville, NY) are shown. γH2Ax foci (red) were counted in the frontal cortex of AD and PF animals using ImageJ (National Institutes of Health). Data are shown as mean ± SE, n = 4–6 animals per group done in duplicate. (TIFF 2702 kb) [file 13148_2017_416_MOESM1_ESM.tiff]

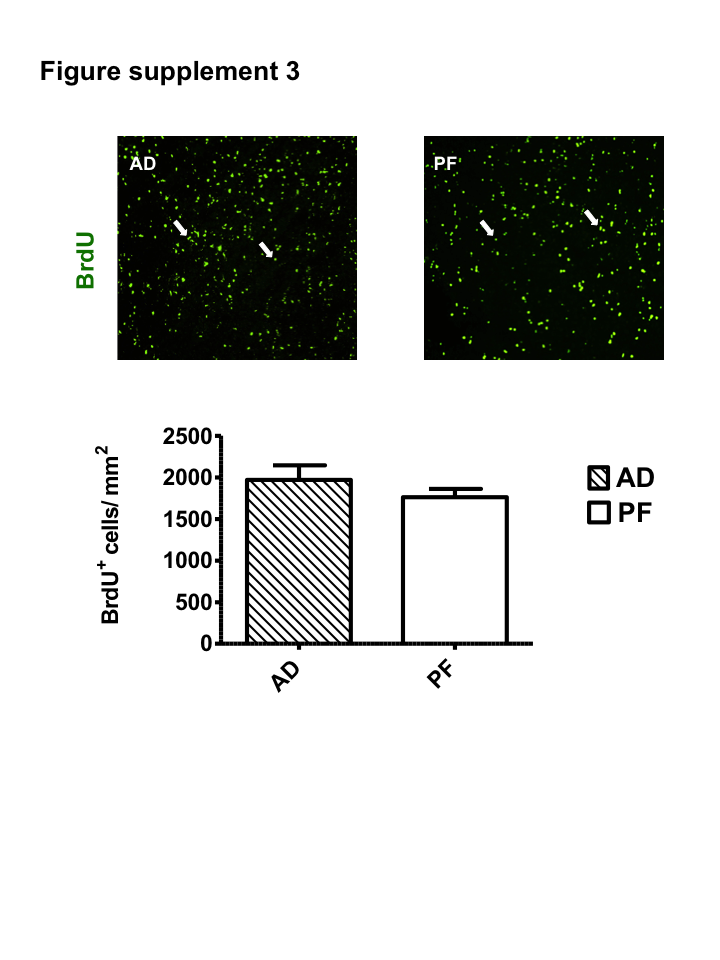

Supplement: Supplementary file 2 — BrdU incorporation and cell proliferation in the frontal cortex of AD and PF control animals. Representative immunofluorescence images, at 20X, visualized using the fluorescence microscope Nikon Eclipse 2000 (Nikon, Melville, NY) are shown. BrdU positive cells (green) were counted in the frontal cortex of AD and PF animals using ImageJ (National Institutes of Health). Data are shown as mean ± SE, n = 4–6 animals per group done in duplicates. (TIFF 2702 kb) [file 13148_2017_416_MOESM2_ESM.tiff]

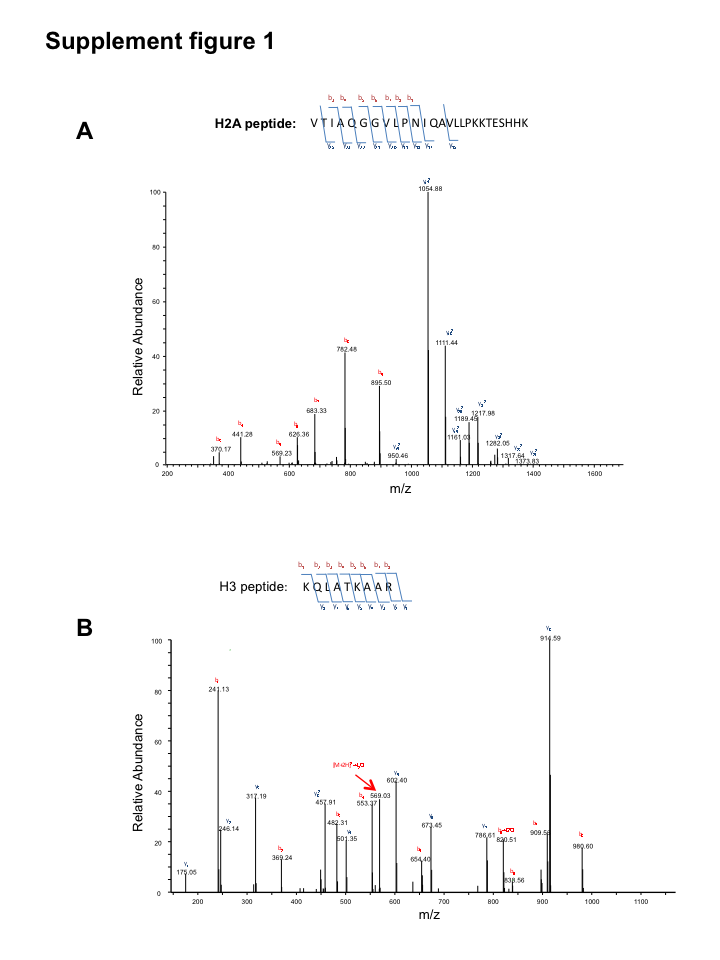

Supplement: Supplementary file 3 — MS/MS spectra for rat histone H2A peptide (vTIAQGGVLPNIQAVLLPkkTESHHk) (A) and histone H3 peptide (kQLATKAAR) (B). Several sequence specific N-terminal (b) and C-terminal (y) ions are identified in these spectra, and these ions confirm the peptides and protein identification. Please note all N-termini and all Lysine residues were propionylated by chemical derivatization. (TIFF 2702 kb) [file 13148_2017_416_MOESM3_ESM.tiff]
