# Supplementary material for: Turnover of histones and histone variants in postnatal rat brain: effects of alcohol exposure
Source: Clin Epigenetics. 2017 Oct 23;9:117. doi: 10.1186/s13148-017-0416-5 (PMC5654083; doi:10.1186/s13148-017-0416-5)
Supplement: Supplementary file 4 — Summary of the effects of PAE on FSR of histones in frontal cortex and hypothalamus of rat brain. FSR is calculated as described in the “Methods” section. Un: unmodified; Ac: acetylated; me2: di-methylation; me3: tri-methylation. Data are shown as mean ± SE, n = 5–8 per group, *p < 0.05, **p < 0.01, ***p < 0.001, AF vs PF, #p < 0.05, ##p < 0.01, ###p < 0.001 Nac vs Ac. (DOCX 16 kb) [file 13148_2017_416_MOESM4_ESM.docx]

**Supplemental table 1:** Summary of histone fractional Synthesis rate (FSR) in frontal cortex and hypothalamus of postnatal rat brain.

|  | **FSR/day in frontal cortex** | | **FSR/day in Hypothalamus** | |
| --- | --- | --- | --- | --- |
| **Histone** | **PF** | **AF** | **PF** | **AF** |
| H2Aun  H2AK120meK126ac | 0.118 ± 0.006  0.100 ± 0.016 | 0.071 ± 0.006******  0.059 ± 0.018 | 0.096 ± 0.005  0.102 ± 0.008 | 0.053 ± 0.005******  0.065 ± 0.014****** |
| H2Bun  H2BK9ac | 0.025 ± 0.008  0.049 ± 0.015 | 0.017 ± 0.006  0.054 ± 0.025 | 0.034 ± 0.001  0.074 ± 0.012**^##^** | 0.011 ± 0.002*******  0.034 ± 0.005****** **^#^** |
| H3un  H3K1ac  H3K9  H3K9me2  H3K9me3  H3K9ac | 0.063 ± 0.005  0.092 ± 0.005**^##^**  0.069 ± 0.004  0.028 ± 0.007  0.030 ± 0.008  0.108 ± 0.007**^#^** | 0.048 ± 0.004******  0.080 ± 0.004**^##^**  0.034 ± 0.005  0.032 ± 0.002  0.015 ± 0.005  0.057 ± 0.016 | 0.049 ± 0.003  0.078 ± 0.002**^#^**  0.035 ± 0.005  0.038 ± 0.006  0.018 ± 0.003  0.066 ± 0.013**^#^** | 0.034 ± 0.002**  0.040 ± 0.013*  0.021 ± 0.001  0.031 ± 0.004  0.009 ± 0.001*******  0.035 ± 0.012 |
| H4un  H4K5acK9acK13ac | 0.043 ± 0.004  0.085 ± 0.007**^##^** | 0.023 ± 0.005******  0.082 ± 0.001**^###^** | 0.024 ± 0.003  0.060 ± 0.015**^#^** | 0.017 ± 0.001*****  0.045 ± 0.008**^#^** |
| H1 | 0.069 ± 0.009 | 0.034 ± 0.009******* | 0.056 ± 0.006 | 0.035 ± 0.002***** |
| H1.1 | 0.178 ± 0.032 | 0.088 ± 0.007***** | 0.122 ± 0.015 | 0.064 ± 0.013* |
| H1.4 | 0.055 ± 0.012 | 0.050 ± 0.005 | 0.062 ± 0.003 | 0.037 ± 0.002******* |
| H1.5 | 0.061 ± 0.010 | 0.034 ± 0.014 | 0.069 ± 0.004 | 0.048 ± 0.001***** |
| H3.3 | 0.165 ± 0.024 | 0.089 ± 0.006****** | 0.075 ± 0.016 | 0.061 ± 0.002 |
| H2Ax | 0.057 ± 0.008 | 0.049 ± 0.005 | 0.046 ± 0.015 | 0.018 ± 0.004***** |
| H2Az | 0.136 ± 0.024 | 0.081 ± 0.019****** | 0.116 ± 0.009 | 0.085 ± 0.0055***** |
